# Surgical treatment of double aortic arch malformation combined with descending aortic arch dissection in adults: a case series

**DOI:** 10.1186/s13019-023-02244-y

**Published:** 2023-05-09

**Authors:** Xinping Min, Zhipeng Hu, Zhiwei Wang, Jun Xia

**Affiliations:** Department of Cardiovascular Surgery, Renmin Hospital of Wuhan University, No. 9 ZhangZhiDong Street, Wuchang District, Wuhan, 430060 Hubei China

**Keywords:** Double aortic arch, Adults, Descending aortic dissection, Surgical treatment

## Abstract

**Background:**

Double aortic arch (DAA) combined with descending aortic arch dissection (DAAD) in adults is a rare aorta vascular disease. Due to the abnormal anatomy of the double arch and arch vessels, the clinical symptoms and surgical methods differ from those of typical aortic dissection.

**Methods:**

This study was retrospective analysis of a case series involving three patients (mean age, 47.3 years) with DAA combined DAAD underwent total arch replacement or hybrid aortic repair from September 2010 to June 2019. The patients’ demographics, initial symptoms, comorbidities, surgical procedures, and outcomes are summarized.

**Results:**

Total arch replacement plus frozen stent implantation under deep hypothermic circulatory arrest was performed for 2 patients, one of them developed disseminated intravascular coagulation and multiple organ failure postoperatively. Case 3 underwent a hybrid procedure with left subclavian artery revascularization and thoracic endovascular aortic repair. The symptoms of hoarseness and dysphagia were obviously improved during the follow up.

**Conclusion:**

In addition to typical sudden chest and back pain, patients with DAA and DAAD may have hoarseness and dysphagia. Based on the development of DAA, total arch replacement or hybrid surgery may be is an optional treatment.

## Introduction

Double aortic arch is a very rare congenital heart disease, it forms a vascular ring, compressing the esophagus and trachea, causing respiratory or gastrointestinal symptoms mainly in infants and young children, symptoms rarely occur after adulthood [1]. It is not clear whether double arch deformity is a risk factor for dissection, and how to perform surgical treatment of DAA combined with DAAD in adult patients is rarely reported. We reviewed 3 patients who underwent surgical treatment in our center and reported as follows.

## Case description

### Case 1

**Fig. 1:**
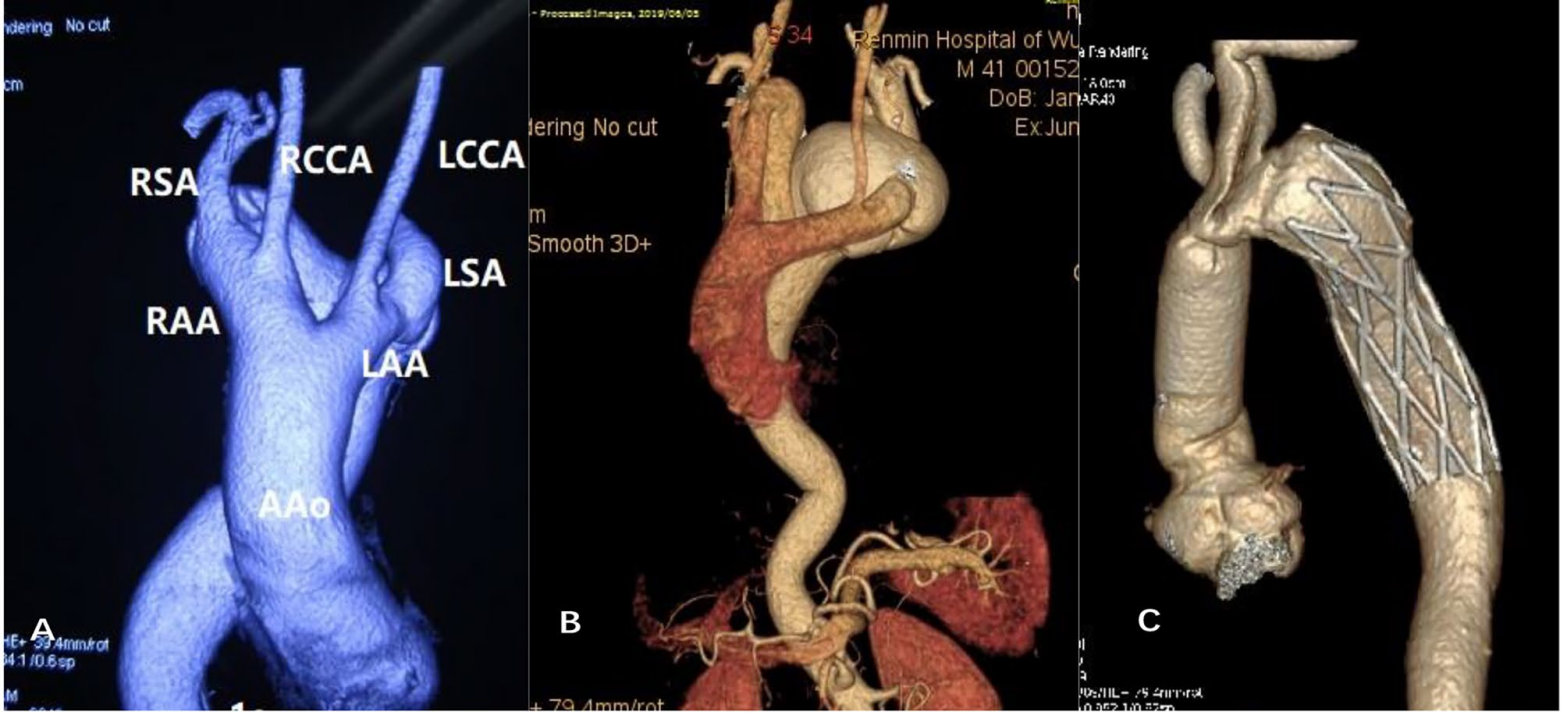
**DAA with descending arch dissection** **A**, DAA with right dominant arch **B**, DAA with left dominant arch **C**, Sun’s procedure for DAA combined with arch dissection AAO, ascending aorta, RAA, right aortic arch; LAA, left aortic arch

, a 46 years old male patient, was hospitalized in September 2010 due to “sudden chest and back pain for two days”. The patient suffered dysphagia for nearly one year. A gated computed tomography angiogram of Aorta (CTA, Fig. 1. A) showed DAA and DAAD. The diameter of the anterior arch was smaller than that of posterior arch (1.5 cm vs. 2.0 cm). The left subclavian artery (LSA) and left common carotid artery (LCCA) originated from the left anterior arch, while the right subclavian artery (RSA) and right common carotid artery (RCCA) originated from the posterior arch.

During deep hypothermic circulatory arrest (DHCA, 25℃), a covered vascular stent (frozen elephant trunk, MicroPort 24 mm * 100 mm) was placed in the true cavity of the descending aorta. The stent was anastomosed end-to-end with the distal end of the bifurcated vessel graft (Terumo 24 mm). The LCCA, RCA and bilateral subclavian arteries were reconstructed successively. Delayed chest closure was selected due to excessive bleeding. Unfortunately, the patient died two days later because of multiple organ failure.

### Case 2

, a 41 years old male patient was transferred to hospital in June 2019 due to “sudden chest and back pain for one day”. He had progressive dysphagia with hoarseness in recent months. Aortic CTA indicated that (Fig. 1.B): DAA, and dissected aneurysm formed in the descending aortic arch. Both left and right arches gave rise a common carotid artery proximally and a subclavian artery distally. The diameter of the anterior arch was larger than posterior arch (1.8 cm vs. 1.3 cm).

During DHCA, a covered stent (MicroPort 26 mm*120mm) was placed in the true lumen of the descending aorta. LSA, LCA and RCA were reconstructed using vascular graft with branches (Terumo 24 mm). Due to its deep location, it was hard to expose and reconstruct RSA (Fig. 1.C). The surgical time of operation, cardiopulmonary bypass, and aortic cross clamp were 7 h, 115 min, and 40 min respectively. During three-year follow-up time, the symptoms of dysphagia and hoarseness were obviously improved.

### Case 3

**Fig. 2:**
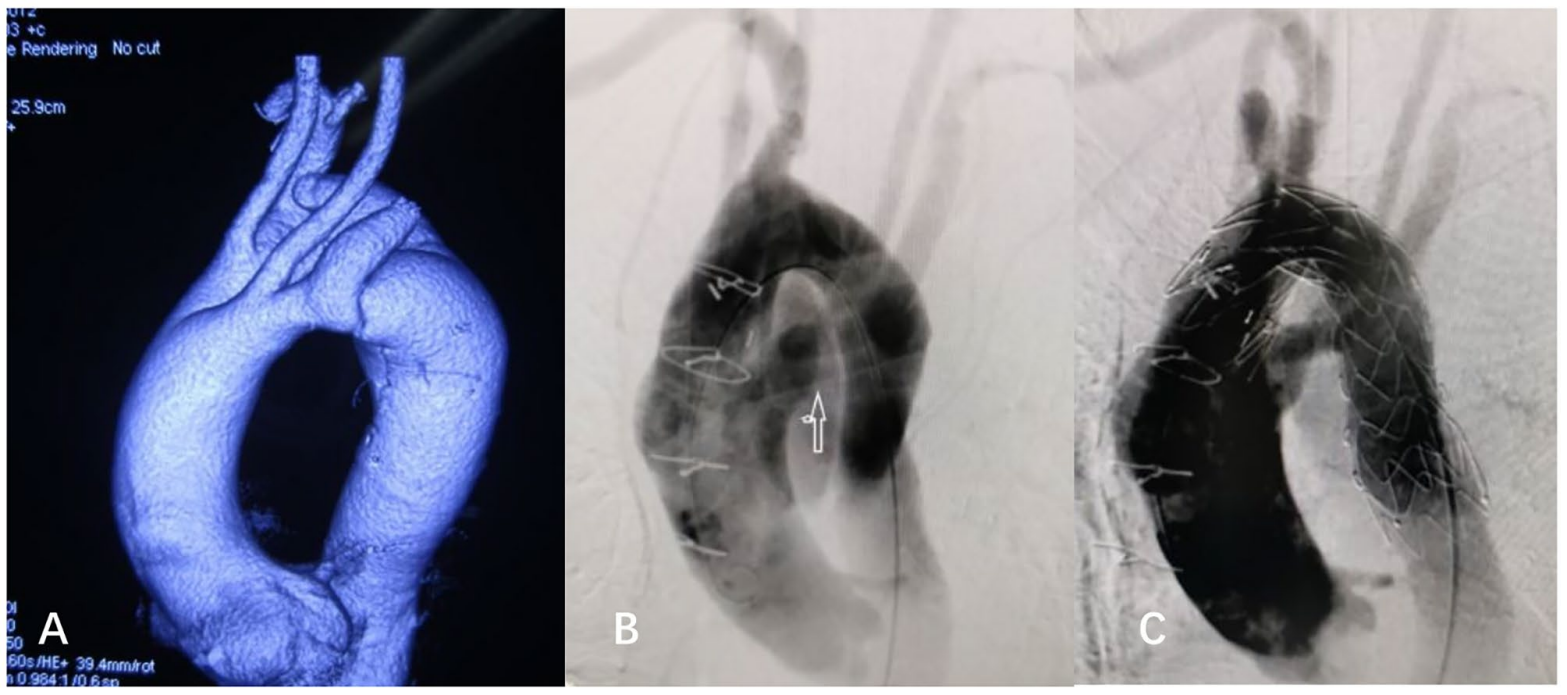
**Combined operation for aortic arch dissection with DAA** **A**, showed DAA with the right dominant arch and dissection at the descending arch **B**, arrow indicated the severed left arch and the reconstructed LSA. **C**, showed a membrane stent sealed the breach of descending arch

a male patient, 55 years old, was admitted to the hospital in October 2012 because of “sudden chest and back pain for one day”, had a history of hypertension without dysphagia and other symptoms. CTA of aorta showed DAA. The right dominant arch (2.2 cm) gave rise RSA and RCCA, and the LSA and LCA arose directly from the anterior arch (1.0 cm) (Fig. 2. A).

Using a hybridization technique, firstly we severed the nondominant anterior arch between LCCA and LSA through medium sternotomy, LSA was anastomosed to LCA end-to-side (Fig. 2. B), and then a covered stent (Medtronic 24 mm*157mm) was delivered to seal the dissection break through the left femoral artery approach (Fig. 2. C). This patient recovered smoothly, and no related complications occurred during follow-up.

## Discussion

This combination of DAA and DAAD represents an association between acquired a congenital arch anomaly and aortic disease, it is known that dissection of the aorta seems to occur with greater frequency in patients with bicuspid aortic valve and coarctation of the aorta, two more common congenital anomalies. As an uncommon arch anomaly, it is likely that DAA is at risk for dissection though infrequently encountered in the adult population.

The operative approach needs to be tailored to fit the anatomy, the difficulty in the treatment of adult patients with DAA and DAAD is the abnormality of arch vessels. The rate of right dominant arch accounts for 75%, and that is 20% for left dominant arch, and 5% for the balanced type [2]. Surgical treatment of related cases is rarely reported in the literature. Toyokawa et al. reported a case of DAA with ascending aortic dissection, with ascending aorta and partial hemiarch replacement [3]. Midulla PS et al. successfully performed descending aortic arch replacement through right thoracotomy for a patient with DAA combined with dissection aneurysm of right descending thoracic aorta [4]. In this case series, because of dissection lesion involving the dominant arch, two patients underwent total arch replacement under deep hypothermic circulatory arrest, meanwhile, a frozen elephant trunk stent was placed in the descending thoracic aorta. In case 3, the proximal end of the dominant arch has sufficient anchoring area and appropriate vessel diameter, therefore, we used a hybrid approach to surgically disconnect the non-dominant arch, reconstruct the left subclavian artery, and repair the thoracic descending aortic lesions.

Presently, the major use of Three-Dimensional printing(3DP) in vascular surgery as reported by literature is offering visual and tactile support for pre-operative planning, although other anecdotal applications are described as well, namely guiding fenestration of endografts and simulating complex endovascular procedures [5]. Research of Tang F et al. [6] demonstrated that it was feasible to rapidly design and manufacture a customized aortic stent graft assisted by 3DP technology, which showed improved geometric compliance and physical characteristics than the straight stent graft. These great advances in Three-Dimensional printing will indefinitely promote endovascular repair for patients with congenital arch anomaly.

## Conclusion

To the best of our knowledge, this is the first case series that reports patients with DAA combined with DAAD, the therapeutic scheme can be selected according to the dominance of the arch and lesion location at the aorta. Hybrid surgery or Sun’s procedure is an available therapeutical method, and endovascular therapy assisted by 3DP technology may be another choice in future.

## Data Availability

All data included in this report are available upon request by contact with the corresponding author.

## Abbreviations

DAA: double aortic arch
DAAD: descending aortic arch dissection
LSA: left subclavian artery
LCCA: left common carotid artery
RSA: right subclavian artery
RCCA: right common carotid artery
DHCA: deep hypothermic circulatory arrest
3DP: Three-Dimensional printing
